# Methodology for Evaluating the CO_2_ Sequestration Capacity of Waste Ashes

**DOI:** 10.3390/ma16155284

**Published:** 2023-07-27

**Authors:** Sara Tominc, Vilma Ducman

**Affiliations:** Laboratory for Cements, Mortars and Ceramics, The Department of Materials, Slovenian National Building and Civil Engineering Institute (ZAG), Dimičeva ulica 12, 1000 Ljubljana, Slovenia; sara.tominc@zag.si

**Keywords:** CO_2_ sequestration, carbonation efficiency, coal ash, wood biomass ash, co-combustion ash, DTA-TG analysis

## Abstract

The concentration of CO_2_ in the atmosphere is constantly increasing, leading to an increase in the average global temperature and, thus, affecting climate change. Hence, various initiatives have been proposed to mitigate this process, among which CO_2_ sequestration is a technically simple and efficient approach. The spontaneous carbonation of ashes with atmospheric CO_2_ is very slow, and this is why accelerated carbonation is encouraged. However, not all ashes are equally suitable for this process, so a methodology to evaluate their potential should be developed. Such a methodology involves a combination of techniques, from theoretical calculations to XRF, XRD, DTA-TG, and the calcimetric determination of the CaCO_3_ content. The present study followed the approach of exposing ashes to accelerated carbonation conditions (4% *v*/*v* CO_2_, 50–55% and 80–85% RH, 20 °C) in a closed carbonation chamber for different periods of time until the maximum CO_2_ uptake is reached. The amount of sequestered CO_2_ was quantified by thermogravimetry. The results show that the highest CO_2_ sequestration capacity (33.8%) and carbonation efficiency (67.9%) were obtained for wood biomass bottom ash. This method was applied to eight combustion ashes and could serve to evaluate other ashes or comparable carbon storage materials.

## 1. Introduction

The concentration of CO_2_ in the atmosphere varies with global temperature but has not exceeded 300 parts per million (ppm) for thousands of years [1]. CO_2_ emissions from fossil fuel industries can be a significant cause of CO_2_ accumulation in the atmosphere, with more than 36 Gt CO_2_/year released globally from fossil fuel combustion, cement production, and other industrial processes [2]. For example, the CO_2_ concentration was 280 ppm at the beginning of the Industrial Revolution but has steadily increased since then, while the use of conventional fossil fuels (i.e., coal, oil, and natural gas) became the main source of energy [3]. In 2016, the atmospheric CO_2_ concentration exceeded 400 ppm [1,4]. Stern et al. [5] predicted that the CO_2_ concentration could rise to over 550 ppm by 2050, and this would mean drastic global warming. CO_2_ emissions have been increasing by about 1% per year, with only temporary decreases in 2020 and 2021 due to the COVID-19 pandemic [6,7,8]. In 2022, the atmospheric CO_2_ concentration returned to 420 ppm, and in 2023, CO_2_ emissions reached a record level [9,10].

Several initiatives have been proposed to address the rising CO_2_ concentrations. A strategy based on CO_2_ capture and utilization (CCU) is considered a promising way to reduce CO_2_ emissions and is in line with the basic principle of industrial ecology, which nearly closes material cycles [11,12]. Besides CCU, carbon capture and storage (CCS) is also considered to be a promising strategy to reduce CO_2_ emissions [11]. One of the CCS methods is storing CO_2_ in former oil or gas reservoirs with impermeable cap rock under high pressure [13]. Biomass has additional CCS potential due to the sequestration of atmospheric CO_2_ in biomass ash, and a sustainable industrial production of bioenergy can reduce CO_2_ emissions and minimize the use of expensive CCS technologies [14]. The combination of both approaches is called carbon capture, utilization, and storage (CCUS) and includes pipelines for transportation; injecting CO_2_ into geological formations for storage; and finally its utilization for fuel, chemical, or material products with the potential to reduce about 19% of the global CO_2_ output by 2050 [9]. The cost of CO_2_ capture reached around USD 60–110/t globally in 2022 and is projected to halve by 2030. Reducing costs would allow an increased implementation of these technologies on an industrial scale [9].

In contrast to existing energy- and cost-intensive carbon capture and storage technologies, CO_2_ mineral sequestration represents a low-tech approach that mainly involves the reaction of CO_2_ with Ca- and Mg-rich minerals and the formation of carbonate minerals, e.g., calcite (CaCO_3_), magnesite (MgCO_3_), and dolomite (CaMg(CO_3_)_2_) [2,13,15]. Spontaneous carbonation with atmospheric CO_2_ (0.04%) is generally very slow, so the natural CO_2_ sequestration is insufficient [15,16,17]. Carbonation can be accelerated by an increased concentration or pressure of CO_2_. Combustion residues react with CO_2_, where the most common component is the strongly basic calcium oxide (CaO; Equation (1)) [18]. Directly binding molecular CO_2_ to CaO is a very slow process, and the reaction is faster in the presence of H_2_O (liquid water or moisture) [15]. Here, CO_2_ first dissolves in H_2_O, dissociates, and reacts with Ca-hydroxide to form CaCO_3_, which has very low solubility in H_2_O (Equations (2) and (3)) [18]. This process changes the chemical and physical properties of the ash.

CaO (s) + CO_2_ (g) → CaCO_3_ (s) (1)

CaO (s) + H_2_O (g) → Ca(OH)_2_ (s) (2)

Ca(OH)_2_ (s) + CO_2_ (g) → CaCO_3_ (s) + H_2_O (l) (3)

CO_2_ mineral sequestration is a method that involves the conversion of CO_2_ into stable mineral carbonates and their subsequent storage. The reaction of CO_2_ with minerals can occur without external energy input [19]. Direct mineral carbonation treatment can be divided into dry (or gas–solid) carbonation under a certain relative humidity and aqueous (or fluid–solid) carbonation, in which mineral wastes are immersed in a liquid mixture injected with gaseous CO_2_ [19]. Direct aqueous carbonation is an efficient method for sequestration, and improved reaction kinetics can be achieved by using additives or maximizing temperature and pressure values. However, the increase in the reaction temperature and the complicated operating process limits its use [20]. It is also expensive compared to dry carbonation [19]. The main advantages of carbonation are the high stability of the main product (CaCO_3_), meaning that CO_2_ is unlikely to be released under normal conditions. The reaction is exothermic and requires no additional energy input. The high availability of ashes as by-products of solid fuel combustion is also an advantage [13,21].

The carbonation process also enables us to lower the values of critical parameters such as alkalinity and solubility, which limit the disposal of wood ash in landfills. As spontaneous carbonation is quite slow and ineffective due to the low relative CO_2_ content in air [22], it can be improved by accelerated carbonation, i.e., direct exposure to CO_2_ in the closed chamber, resulting in a higher carbonation rate. The rate of the accelerated carbonation reaction can be determined by the reactivity of CO_2_, which is influenced by the carbonation conditions, such as the particle size of the material (i.e., reaction interface), relative humidity (RH), temperature, contact time, pressure, and CO_2_ concentration [22,23,24]. The applicability of accelerated carbonation depends on the choice of appropriate exposure conditions [16]. In this context, the RH condition is essential to the reaction kinetics. Studies have shown that the reaction rate and the conversion rate of Ca(OH)_2_ increase with increasing RH (up to 91%) [25,26]. Thus, complete carbonation can be achieved at a high RH.

Carbonation is one of the most discussed research topics in the cement and concrete industry. Almost all cement-based materials must undergo a carbonation reaction during their lifetime due to the CO_2_ content in earth’s atmosphere [16]. When CO_2_ diffuses into the concrete, carbonation occurs, and the pH of the concrete decreases to about 9–10. At such a relatively low pH, the existing passive protective layer on the steel surface breaks down and corrosion occurs [17]. Thus, the carbonation reaction of concrete reduces the durability of such materials, while the carbonation reaction in alkali-activated materials (AAMs) poses an even greater risk; it disintegrates the binder matrix and, thus, reduces its strength [16]. However, studies show that using 10% carbonated fly ash as a partial cement replacement is comparable to the early compressive-strength results of mortars without fly ash [15,27]. After accelerated carbonation curing, a 15 kg concrete block (with 13% cement) can store up to 0.47 kg of CO_2_, assuming 24% CO_2_ uptake by the cement [16,28]. Accordingly, carbon emissions from the cement industry could be reduced by 2.5% at the same carbon uptake rate if the cement industry used carbonation curing [28].

Using CO_2_ sequestration for the production of building materials may be the only economically sustainable carbon-negative industrial process and, therefore, deserves to be the focus of further development [18]. A promising option is the replacement of cement in specific applications with alternative binders, using carbstone technology, a new method that is much more environmentally friendly, as no cement or concrete products are used [29,30]. Ghent is the first Belgian city to build a circular footpath with clinkers produced using the carbstone technology. Bricks are produced by reacting residues from the steel industry (steel slag) with CO_2_. The carbstone technology is based on the reaction of Ca- and Mg-containing minerals with CO_2_ to form carbonate binders [30]. Other potential sources from waste streams are ashes containing Ca and Mg compounds, which allow for the sequestration of CO_2_ in the form of carbonate compounds [15,31]. Therefore, wood biomass ash (WBA) is an attractive candidate for CO_2_ sequestration as a Ca-rich ash [13,32]. In total, 70% of WBA is still landfilled, although it is a potential substitute material in various construction sectors or for CO_2_ sequestration [11,13,15,33].

Although CO_2_ sequestration is a promising carbon capture and storage technology, not much is known about the sequestration potential of various waste ashes. Tamiselvi Dananjayan et al. [34] reported the highest carbonation efficiency (CE) of coal fly ash (67.87%) achieved by the aqueous carbonation route under optimized process conditions (4 bar pressure with 100% CO_2_). Liu et al. [20] reported a sequestration efficiency of 28.74% of fly ash in the circulating fluidized bed process with the addition of 20% H_2_O (g) by direct gas–solid carbonation, while Koch et al. [13] determined a low average CE of 16.44% for bottom wood ash at different mixing ratios with water in batch experiments.

The main objective of the work presented here is to provide a methodology for evaluating the sequestration capacity of (Ca-rich) combustion waste ashes by a one-step direct gas–solid carbonation process with controlled CO_2_ concentration, temperature, and RH. Using a number of complementary analytical methods, including the mass gain method; calcimetric measurements; and DTA-TG, XRF, and XRD analyses, the degree of carbonation can be confirmed, and the maximum carbonation efficiency can be calculated, allowing the sequestration potential of each ash to be determined. 

## 2. Materials and Methods

### 2.1. Materials

**Figure 1:**
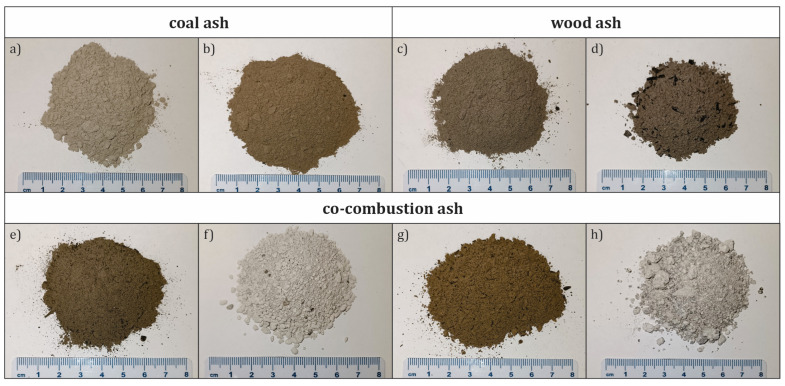
Waste ashes from different sources: (**a**,**b**) fly ash from coal (A1 and A2), (**c**) fly ash (A3) and (**d**) bottom ash from wood (A4), (**e**) fly ash (A5) and (**f**) mixed ash (A6) from a paper mill, and (**g**) fly ash (A7) and (**h**) bottom ash (A8) from a Slovenian heating and power station.

Eight ashes were studied (Figure 1), including two coal fly ashes from German (A1) and Slovenian thermal power plants (A2). Two other ashes (A3 and A4) originated from a Slovenian heat power station, with A3 representing fly ash and A4 bottom ash produced from the combustion of wood biomass (wood chips). Two more ashes came from a paper mill, where the fuel source for A5 fly ash is coal, biomass, and paper sludge, while the fuel source for A6 was fiber paper sludge, waste wood, and bark, resulting in a mixture of 90% bottom ash and 10% fly ash. The last two ashes were from a Slovenian combined heat and power station, where the fuel source is brown coal and wood biomass–wood chips (15%); A7 is fly ash, while A8 is bottom ash.

### 2.2. Characterization

All ashes were homogenized by quartering, packed in a PVC bag, and stored in a plastic container.

The ashes were sieved below 63 µm and placed in 27 mm holders for mineralogical analyses, which were performed before and after CO_2_ exposure by X-ray diffraction (XRD; Empyrean X-ray Diffractometer, Cu X-ray source; PANalytical, Almelo, The Netherlands) in 0.013° steps from angles of 4–70°, under clean room conditions, using the external standard method (corundum NIST SRM 676a). Mineral analyses were performed using the PANalytical X’Pert High Score Plus diffraction software v. 4.8.

The ashes were sieved below 125 µm for chemical analyses, dried at 105 °C, and heated at 950 °C to determine their loss on ignition (LOI); a fused bead was then prepared with a mixture of ash and flux (50% lithium tetraborate/50% lithium metaborate) in a 1:10 ratio (0.947 g: 9.47 g) and heated at 1100 °C. The standard deviation of repeatability for LOI is 0.04 mass percent according to the standard EN 196-2:2013 [35]. The chemical composition of the ashes was the determined using a ARL PERFORM’X Wavelength Dispersive X-Ray Fluorescence Spectrometer (WDXRF; Thermo Fischer Scientific Inc., Ecublens, Switzerland) with an Rh-target X-ray tube and the UniQuant 5 software (Thermo Fisher Scientific Inc., Walthem, MA, USA). The free CaO content was determined according to the standard EN 451-1:2017 [36]. Two measurements were performed for each ash. A mixture of butanoic acid, 3-oxo-ethyl ester, and butan-2-ol was added to the weighed, homogenized sample, sieved under 63 µm. The flask was fitted with the spiral reflux condenser and the absorption tube and boiled for 3 h. The warm mixture was filtered through a filter crucible, and the residue was washed with propan-2-ol. A few drops of bromophenol blue indicator were added to the filtrate, and the filtrate was titrated with HCl until the color changed to yellow. The free CaO content was expressed as a mass percent of dry ash. The standard deviation of repeatability for the determination of free CaO content is 0.03 mass percent.

The CO_2_ sequestration capacity of all ashes was first tested at a controlled RH of 50–55% and a temperature of 20 ± 1 °C and then at an elevated RH of 80–85%. The received ashes were sieved to a grain size below 125 µm and exposed to 4 ± 0.1 vol% CO_2_ for 28 days in a closed carbonation chamber. Samples were taken after 1, 7, 14, 21, and 28 days. The carbonated samples were dried at 105 °C for 24 h for further analyses.

The dried and sieved carbonated ashes were analyzed using a pressure calcimeter (OFITE Calcimeter, OFI Testing Equipment Inc., Houston, TX, USA, according to ASTM D 4373) with an analytical error of <5%. In the OFITE Calcimeter, CaCO_3_ reacted with 10% HCl in a closed reaction cell to form CaCl_2_, CO_2_, and H_2_O. The pressure of the released CO_2_ was measured with a manometer. The calcimeter was calibrated by reacting HCl with pure CaCO_3_ before the actual measurements.

A differential thermal and thermogravimetric analysis (DTA-TG) was performed on carbonated ashes, using a STA 409 PC Luxx Simultaneous thermal analyzer (Netzsch-Gerätebau GmbH, Selb/Bayern, Germany) from 25 to 1000 °C, with a heating rate of 10 K min^−1^. Prior to the measurements, the ashes were dried at 105 °C and sieved below 63 µm. To prevent oxidation during the measurement, the sample chamber was filled with N_2_ with a flow rate of 20 mL min^−1^. Ash batches with an initial mass of 50 mg were placed in Al_2_O_3_ crucibles. TG measured the weight loss in the temperature range of decomposition of the carbonate mineral (550–950 °C), with an analytical error of <1%. The results were analyzed using the Proteus Thermal Analysis software v. 5.2.0 (Netzsch-Gerätebau GmbH, Selb/Bayern, Germany) Each ash was analyzed separately.

The specific surface area was determined by using the Brunauer–Emmett–Teller (BET) method according to ISO 9277.2010, with a Micromeritics ASAP-2020 analyzer (Micromeritics, Norcross, GA, USA) The ash particle shape and size were determined by scanning electron microscopy (a JEOL IT500 LV SEM, Tokyo, Japan) The uncoated ash was placed on the holder and examined in low vacuum mode, using an accelerating voltage of 20 kV and a working distance of 10 mm.

### 2.3. CO_2_ Sequestration Capacity and Carbonation Efficiency

The theoretical maximum of sequestered CO_2_ was determined based on the chemical composition of the ashes in weight percent (wt%), using the Steinour stoichiometric formula (Equation (4)), as reported in the literature [13,16,23,37]:

CO_2-max_ (wt%) = 0.785 (CaO − 0.7 SO_3_) + 1.091 MgO + 0.935 K_2_O + 1.420 Na_2_O (4)

The mass uptake (mass_-uptake_) was determined using the mass gain method, where m_ash_ is the original mass of the waste ash before the carbonation treatment, and m_CO2_ is the mass of the CO_2_ sequestered during the carbonation treatment (Equation (5)) [15]. The CO_2_ sequestration capacity was determined using TG analysis. The carbonation efficiency (CE) was determined as the ratio between the percentage of CO_2_ sequestered experimentally according to the TG analysis and the theoretical CO_2_ value calculated using Steinour’s equation (Equation (6)) [13]. Higher carbonation efficiency indicates better CO_2_ sequestration [13].

mass_-uptake_ (%) = m_CO2_/m_ash_ × 100 (5)

CE (%) = CO_2-TG exp_ (%)/CO_2-max_ (%) (6)

## 3. Results and Discussion

### 3.1. Chemical and Mineralogical Composition of the Analyzed Ashes

**Figure 2:**
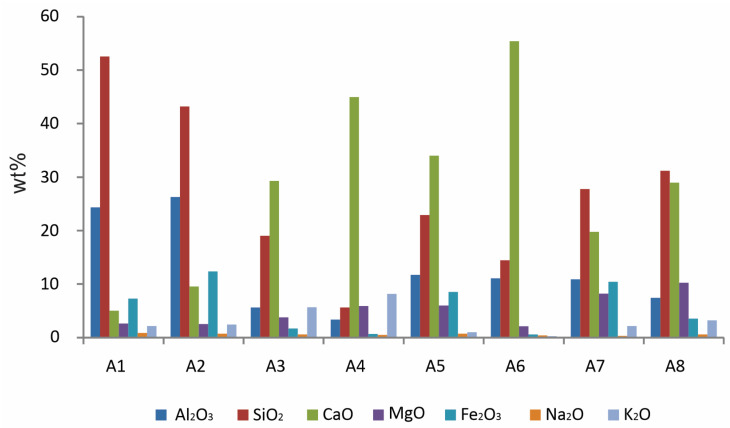
Comparison of the primary oxides of the studied ashes by XRF analysis.

The XRF analysis of the ashes A1 and A2 showed that the coal ashes mainly consist of SiO_2_, Al_2_O_3_, Fe_2_O_3_, and CaO, while the other ashes had a significantly higher wt% content of CaO (especially A4, A5, and A6) and lower contents of Al_2_O_3_, Fe_2_O_3_ (except A7), and SiO_2_. The WBAs (A3 and A4) mainly consist of CaO, Al_2_O_3_, K_2_O, MgO, and SiO_2_, as shown in Figure 2. The ashes A3-8 have high alkali metal contents, such as Ca and Mg, which are favorable for CO_2_ sequestration. The mean values of the primary oxides measured by XRF and the LOI at 550 and 950 °C are given in Table 1.

Analyzing the crystal phases before the CO_2_ exposure showed that the coal ashes mainly contain quartz, mullite, magnesioferrite, hematite, and anorthite, whereas WBA mainly contains calcite, lime, portlandite, fairchildite, and quartz. The co-combustion ashes contain calcite, lime, quartz, anhydrite, periclase, and minor phases of portlandite, hematite, brownmillerite, akermanite, and larnite. According to the literature, possible carbonation minerals are Ca and Mg oxides (lime and periclase), hydroxides (portlandite), sulfates (anhydrite), and silicates [38,39].

**Figure 3:**
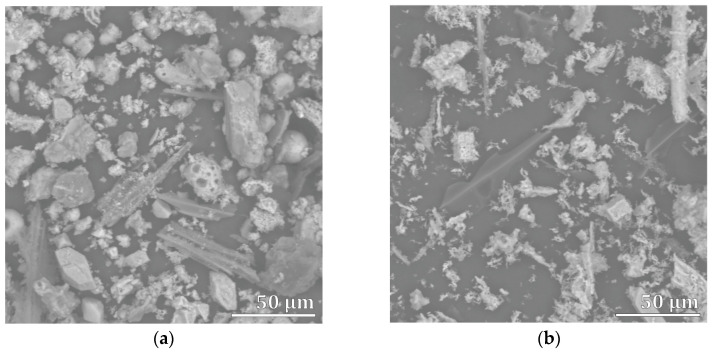
SEM micrographs of (**a**) wood biomass fly ash (A3) and (**b**) wood biomass bottom ash (A4).

The physical and chemical properties of ash are significantly affected by the type of combustion technology from which it originates [40]. In our study, fly ash from wood biomass (A3) had a much lower specific surface area than bottom ash from wood biomass (A4; Table 1), which was also confirmed by SEM (Figure 3). Larger particles are generally considered to be less reactive to CO_2_ and have a lower specific surface area [22,41]. In our case, the ashes A4, A5, and A7 had a much larger specific surface area than the others, which could affect their reactivity to CO_2_.

The approach adopted in this study is the so-called “reverse” analysis. All received ashes, even directly from the production line, but especially if stored for some time, already experienced a certain degree of carbonation. Therefore, it would be incorrect to base the calculations on the carbonate content at the beginning of the measurements and after the exposure. Instead, the complete carbonation must be addressed, and then the maximum potential for CO_2_ sequestration must be evaluated.

**Figure 4:**
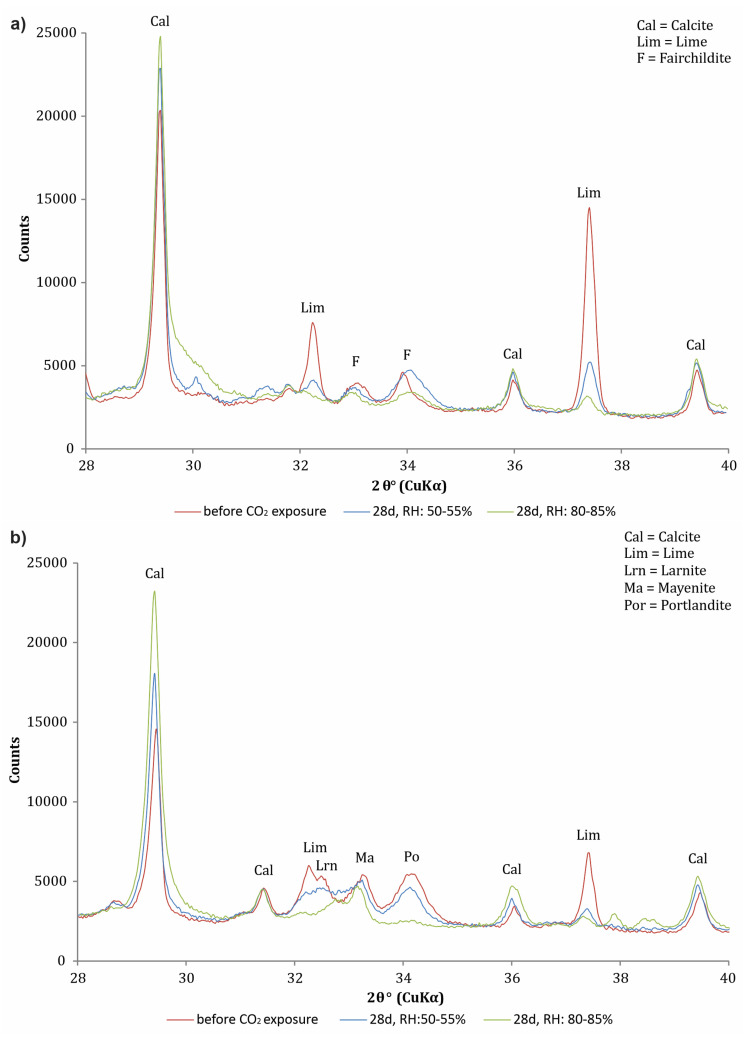
X-ray diffractograms of (**a**) A4 and (**b**) A6 before and after 28 days of CO_2_ exposure at an RH of 50–55% and 80–85%, respectively. The main peaks of calcite (Cal) are marked at 2θ ≈ 29.4°, 36.0°, and 39.4°; and for lime (Lim), they are marked at 2θ ≈ 32.2° and 37.4°.

According to the analysis of the crystalline phases, the most obvious differences between the X-ray diffractograms of the ashes after 28 days of CO_2_ exposure were in the presence of calcite and lime peaks (Figure 4). After 28 days of CO_2_ exposure and an RH of 80–85%, the intensity of the lime peaks decreased significantly. A similar drop in intensity is observed for ash A6. In both samples (A4 and A6), calcite is already present before CO_2_ exposure, meaning that the ash is already partially carbonated. However, with exposure to CO_2_ and an increase in RH (80–85%), the intensity of the calcite peaks increases significantly. Thus, the XRD analysis allows us to follow the carbonation process and the relative content of the calcite/lime.

We also observed other possible carbonation minerals, such as portlandite (Ca(OH)_2_). Many authors have studied the carbonation of portlandite at different RHs and found that the carbonation of portlandite can be avoided at a low RH, while at a very high RH (>90%), the water film on the surface of portlandite consists of a larger number of water monolayers, allowing CaCO_3_ to nucleate in the aqueous layer and at the crystal/solution interface [25,26,42,43]. In addition to a faster reaction, complete carbonation can be achieved at a high RH [26], and we were able to confirm this with our X-ray diffractograms. After 28 days of CO_2_ exposure, portlandite is no longer detectable at an RH of 80–85%, while at an RH of 50–55%, the peak is still very prominent (Figure 4b).

### 3.2. Calcimetric Measurements

**Figure 5:**
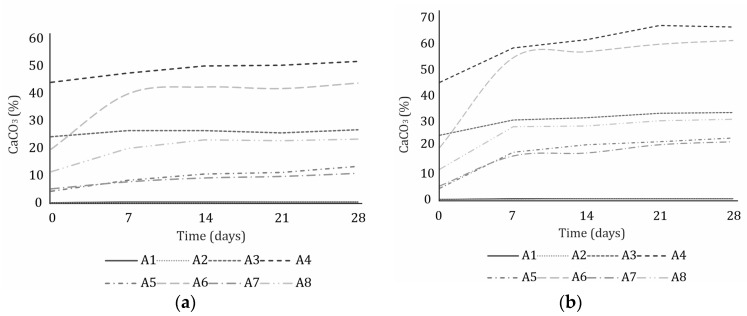
CaCO_3_ content in ashes A1-8 during 28 days of CO_2_ exposure in the carbonation chamber (**a**) at an RH of 50–55% and (**b**) 80–85%.

The trend of increasing CaCO_3_ content was monitored by calcimetric measurements. Figure 5 shows that the CaCO_3_ content increased rapidly during the first week of CO_2_ exposure and then increased very slowly. When the CaCO_3_ content stops increasing, maximum carbonation is reached. After 28 days of CO_2_ exposure at an RH of 50–55%, the curve for ashes A4 and A6 still increased slightly, indicating that maximum carbonation was not yet reached for these ashes. The calcimetric measurements are consistent with the X-ray diffractograms in Figure 4, where the lime and portlandite peaks are still clearly visible after 28 days of CO_2_ exposure at an RH of 50–55%. However, when the RH was increased to 80–85%, the CaCO_3_ content stopped increasing (Figure 5b), indicating that maximum carbonation was achieved.

### 3.3. DTA-TG Analysis

**Figure 6:**
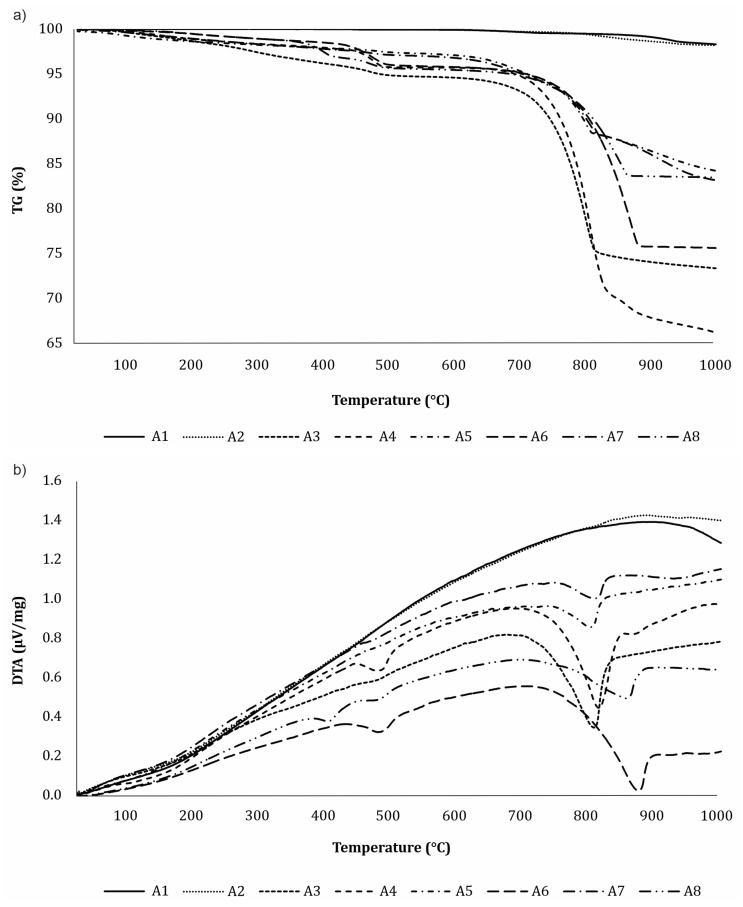
(**a**) TG analysis and (**b**) DTA analysis for carbonated ashes after 28 days of CO_2_ exposure at an RH of 50–55%.

**Figure 7:**
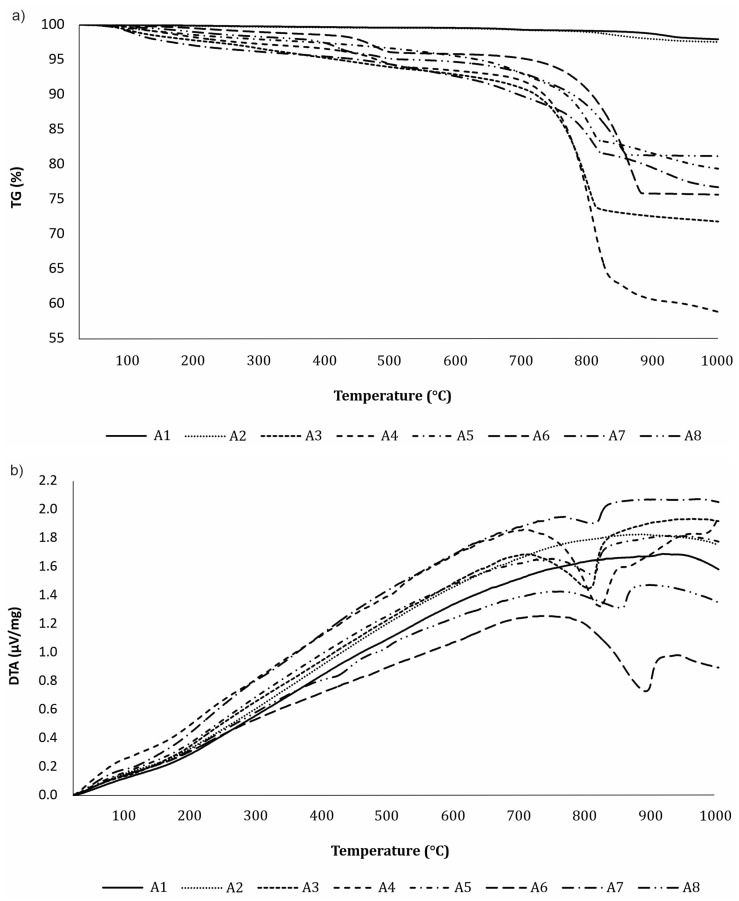
(**a**) TG analysis and (**b**) DTA analysis for carbonated ashes after 28 days of CO_2_ exposure at an RH of 80–85%.

Thermogravimetry (TG) measures the mass change of a sample when it is subjected to a temperature program in a controlled atmosphere [44]. Because of possible hydration reactions contributing to the weight gain, TG is a suitable method to quantify the increase in the CaCO_3_ content during carbonation and provides quantitative information on the extent of carbonation [16,22]. Figure 6 shows the (a) TG and (b) DTA analysis for carbonated ashes after 28 days of CO_2_ exposure at RH of 50–55%, and Figure 7 at RH of 80–85%. The weight loss measurements in the temperature range of 550–950 °C provide information about the amount of sequestered CO_2_.

Three main peaks can be observed on the DTA diagram. The first peak, which is below 200 °C, can be attributed to evaporable water in the ash, but it is not prominent because the ash was dried before analysis. The second peak, at about 400–500 °C, is caused by the dehydration of portlandite [37]. The portlandite peak is the most visible in ashes 4, 6, and 8 after CO_2_ exposure at lower RHs (Figure 6b), while this peak is no longer visible after CO_2_ exposure at higher RHs (Figure 7b), as is consistent with previous XRD analyses. The third peak, which contributes to the mass loss in the temperature range from 550 °C to 950 °C, results from the decomposition of CaCO_3_ into CaO and the release of CO_2_ (Equation (7)). The weight loss in this range is higher for carbonated samples, thus confirming that the carbonation process was successful [34].

CaCO_3_ (s) → CaO (s) + CO_2_ (g) (7)

A DTA analysis can distinguish the decomposition of portlandite and calcite, a task which is not possible using the mass gain method. The CaCO_3_ decomposition peak was observed in all ashes except the coal fly ashes (A1 and A2), indicating that these ashes have no potential for CO_2_ sequestration, as expected. After 28 days of CO_2_ exposure at an RH of 80–85%, the biomass ashes A3 (21.3%) and A4 (33.8%) and mixed ash from the paper mill A6 (28.8%) had the greatest CO_2_ sequestration potential of the analyzed ashes. Both the fly ashes from the paper mill (A5) and from the co-combustion of brown coal and biomass wood chips sequester 15.9% of CO_2_, while bottom ash from the Slovenian heating and power station (A8) sequesters 13.7%. At a lower RH, the CO_2_ sequestration capacity is slightly lower for all ashes due to incomplete carbonation (Table 2).

In general, the total CO_2_ release depends on the ash type. Ashes with a higher CaO content in their chemical composition (see Table 1) have a greater CO_2_ sequestration potential, which is also reflected in a higher mass loss in the TG diagram.

### 3.4. CO_2_ Sequestration Capacity and Carbonation Efficiency (CE)

**Figure 8:**
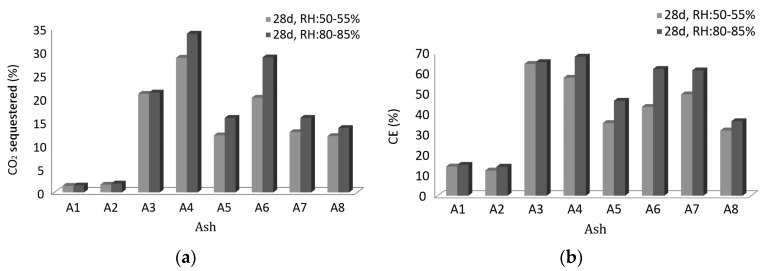
(**a**) Percentage of sequestered CO_2_ determined by TG after 28 days of CO_2_ exposure at RH of 50–55% and 80–85% and (**b**) calculated carbonation efficiency at RH of 50–55% and 80–85% for all ashes.

The CO_2_ sequestration capacity and calculated carbon efficiency for all ashes after 28 days of accelerated carbonation in a 4% CO_2_ environment at RH of 50–55% are presented in Table 2 and at an RH of 80–85% in Table 3. The highest percentage of sequestered CO_2_ (33.8%) and mass uptake (17.3%) was found in bottom ash from wood biomass (A4), which corresponds to a CE of 67.9%, as presented in Table 3 and Figure 8. This means that the ash A4 can sequester 67.9% of the maximum theoretical mass by using the Steinour stoichiometric formula [13,16,23,37]. For fly ash from wood biomass (A3), 65.2% CE was achieved, with 21.3% sequestered CO_2_, while the theoretical maximum of sequestered CO_2_ was 32.6%. However, a minimum mass uptake was measured here, indicating that the ash was already carbonized, probably due to a long-term natural carbonation before sampling [15]. The storage and transport conditions of WBA largely determine the amount of CaO and other carbonates, as carbonation and hydration can occur during these processes under moist conditions. Upon contact with water/moisture, up to 50% of the CaO content of WBA reacts to form Ca(OH)_2_, which, in turn, reacts to CaCO_3_ upon contact with CO_2_ [40].

The mixed ash from the paper mill (A6) sequesters 28.8% of CO_2_, resulting in the highest mass uptake (20.4%) and a CE of 61.9% (Table 3). The CE for fly ash from the same paper mill (A5) was 46.3%. Although A6 is 90% bottom ash and has a broad particle size distribution [45], it is very reactive to CO_2_, contradicting the claim that the fine fly-ash particles are more reactive to CO_2_ than bottom ash [23]. The particle size of an ash should not always be considered a limiting factor for CO_2_ sequestration, but, in general, ashes with a similar chemical composition have a higher carbonation efficiency at smaller particle sizes [15,23,46]. However, if the calcium content of the feedstock varies, the particle size or surface area may be of secondary importance [32].

The presented approach is suitable for waste ashes with a high Ca content. Both types of coal fly ash (A1 and A2) showed neither sequestration potential nor high carbonation efficiency. Nevertheless, the average CE after 28 days of CO_2_ exposure at an RH of 80–85% was 66.6% for WBA, which is much higher than in the study reported by Koch et al. [13]. Most WBA is still landfilled, and disposal costs are high, so a direct use of the ash for CO_2_ sequestration would be beneficial in terms of a circular economy [13].

In further studies, it would be optimal to shorten the processing time by optimizing the carbonation conditions; however, these results open the possibility for future studies using the proposed methodology. To quantify the environmental impact, a life-cycle analysis could also be the subject of future studies.

## 4. Conclusions

This study focused on determining the sequestration potential of the waste ashes in a CO_2_ direct mineral carbonation process using a number of complementary analytical methods. To understand the reaction kinetics, we determined the appropriate conditions for accelerated carbonation. Using XRD and DTA, we followed the carbonation of portlandite and lime at different RHs (50–55% and 80–85%) and monitored the CaCO_3_ content with calcimetric measurements. The sequestration capacity was determined by TG, and the carbonation efficiency was calculated for each ash.

The obtained results showed that the reaction rate increased with increasing RH, and the maximum carbonation was achieved after 28 days in a dense carbonation chamber with 4% *v*/*v* CO_2_, a temperature of 20 °C, and an RH of 80–85%. Ca-rich waste ashes were successfully used for direct mineral carbonation, with the highest carbonation efficiency (67.9%) and sequestration capacity (33.8%) achieved with wood biomass bottom ash (A4). In addition, we showed that wood ash is highly reactive to CO_2_, so its direct use for CO_2_ sequestration would be beneficial in terms of a circular economy because 70% of wood biomass ash is still landfilled.

## Figures and Tables

**Table 1:** Specific surface area (BET), content of free CaO, loss on ignition (LOI) at 550 and 950 °C, and chemical composition of the original ashes in terms of the primary oxides (wt%), measured using XRF.

Ash Type:	Sample ID	Free CaO (%)	BET (m^2^/g)	LOI _550 °C_	LOI _950 °C_	Δ LOI (950–550 °C)	Al_2_O_3_	SiO_2_	CaO	MgO	Fe_2_O_3_	Na_2_O	K_2_O	P_2_O_5_	TiO_2_	SO_3_
Coal ash	A1	0.29	1.34	1.81	2.39	0.55	24.35	52.53	5.03	2.60	7.30	0.86	2.15	0.69	0.88	0.43
	A2	0.42	2.56	0.64	0.64	0.00	26.29	43.20	9.56	2.54	12.37	0.74	2.44	0.34	0.73	0.61
Wood ash	A3	4.61	3.80	13.91	29.91	16.00	5.62	19.04	29.27	3.78	1.70	0.56	5.65	2.24	0.37	1.00
	A4	16.74	41.83	2.26	26.09	23.83	3.35	5.64	44.95	5.88	0.68	0.48	8.14	2.82	0.07	0.32
Co-combustion ash	A5	8.06	41.29	5.20	11.71	6.51	11.71	22.90	34.01	5.99	8.53	0.74	0.99	0.28	0.73	1.70
	A6	20.63	6.36	0.00	14.55	14.55	11.08	14.45	55.40	2.12	0.56	0.41	0.25	0.26	0.22	0.20
	A7	5.11	86.07	11.68	16.98	5.30	10.87	27.78	19.77	8.21	10.44	0.28	2.16	0.56	0.52	1.69
	A8	13.24	5.75	0.38	12.79	12.41	7.43	31.17	28.98	10.25	3.56	0.57	3.21	1.00	0.32	0.07

**Table 2:** Mass uptake (mass_-uptake_)_,_ CO_2_ sequestration capacity determined by TG analysis (CO_2-TG exp_)_,_ theoretical maximum of sequestered CO_2_ according to the Steinour equation (CO_2-max_), and calculated carbonation efficiency (CE) after 28 days of exposure at RH of 50–55%.

Ash	mass_-uptake_ (%)	CO_2-TG exp_ (%)	CO_2-max_ (%)	CE (%)
A1	0.1	1.4	9.8	14.2
A2	0.2	1.6	13.3	12.3
A3	1.7	21.0	32.6	64.4
A4	9.0	28.7	49.8	57.6
A5	5.9	12.2	34.3	35.4
A6	9.9	20.2	46.5	43.3
A7	4.2	12.9	26.0	49.5
A8	5.5	12.0	37.7	31.8

**Table 3:** Mass uptake (mass_-uptake_), CO_2_ sequestration capacity determined by TG analysis (CO_2-TG exp_), theoretical maximum of sequestered CO_2_ according to the Steinour equation (CO_2-max_), and calculated carbonation efficiency (CE) after 28 days of exposure at RH of 80–85%.

Ash	mass_-uptake_ (%)	CO_2-TG exp_ (%)	CO_2-max_ (%)	CE (%)
A1	0.2	1.5	9.8	15.0
A2	0.5	1.9	13.3	14.2
A3	5.0	21.3	32.6	65.2
A4	17.3	33.8	49.8	67.9
A5	11.8	15.9	34.3	46.3
A6	20.4	28.8	46.5	61.9
A7	11.1	15.9	26.0	61.2
A8	7.6	13.7	37.7	36.4

## Data Availability

The data presented in this study are openly available from the repository DiRROS: http://hdl.handle.net/20.500.12556/DiRROS-16698 (accessed on 20 July 2023), and upon request from the first and corresponding author.
